# Ischemic Stroke–A Scientometric Analysis

**DOI:** 10.3389/fneur.2022.893121

**Published:** 2022-04-28

**Authors:** Dominic Millenaar, Andreas Ragoschke-Schumm, Tobias Fehlmann, Maximilian Raible, Piergiorgio Lochner, Michael Böhm, Klaus Fassbender, Andreas Keller, Felix Mahfoud, Christian Ukena

**Affiliations:** ^1^Department of Internal Medicine III, Cardiology, Angiology, Intensive Care Medicine, Saarland University Hospital, Homburg, Germany; ^2^Department of Neurology, Saarland University Hospital, Homburg, Germany; ^3^Chair for Clinical Bioinformatics, Saarland University, Saarbrücken, Germany; ^4^Department for Neurobiology, Stanford University, Stanford, CA, United States; ^5^Institute for Medical Engineering and Science, Massachusetts Institute of Technology, Cambridge, MA, United States

**Keywords:** stroke, ischemic, hemorrhagic, research, citation analysis

## Abstract

**Background:**

Stroke is the second leading cause of death world-wide. A comprehensive scientometric study regarding ischemic stroke research has not been performed yet. This study aims at investigating the global research output on ischemic stroke research.

**Methods:**

All 21,115 articles regarding ischemic stroke were retrieved from the Web-of-Science-Core-Collection and analyzed regarding regional differences, the authors' sex, subtopics of stroke, as well as international research collaborations.

**Results:**

A total of 132 different countries participated, with the USA contributing most publications with 4,614 (21.9%), followed by China with 3,872 (18.3%), and Germany with 1,120 (5.3%). Analyzing the scientific quality of different countries by H-index, the USA ranked first with an H-index of 202, followed by Germany (H-index 135) and the United Kingdom (UK;H-index 129). The most frequently used topic was “Clinical Neurology” with 9,028 publications. Among all first authors attributed to their sex, 32.3% of all first authors were female and 67.7% were male (4,335 vs. 9,097). The proportion of female last authors was comparatively lower at 22.4% (3,083 publications) compared with 77.6% male authors (10,658 publications). There was a broad network of international collaborations.

**Conclusions:**

Research in ischemic stroke has substantially increased over time. Scientists from the USA have the highest number of publications, followed by China and Germany. Measured by the H-index, the USA held the highest publication quality, followed by Germany and the UK. The scientific landscape was male-dominated with 67.7% of all first authors being male. Worldwide international collaborations play a major role in ischemic stroke research.

## Introduction

Stroke is the second leading cause of death world-wide (1). Up to 50% of stroke survivors are chronically disabled, thus causing immense consequences for the economy as well as public health in general (2). Between 2015 and 2018, around 7.6 million individuals aged ≥20 years in the United States of America (USA) were diagnosed with stroke, the majority of which (87%) are ischemic (3). Ischemic strokes are further divided into lacunar and non-lacunar infarcts, the latter also into various subgroups such as cardioembolic, cryptogenic, or occlusion of a major artery (4). In ~17% of all patients with ischemic strokes, the exact cause remains elusive [so called embolic stroke of undetermined source (ESUS)] (5).

Scientometrics is a research area that deals with the research and measurement of scientific literature. It can be used to measure the impact of individual authors, institutes, or even countries within a defined topic area (6). A scientometric analysis regarding stroke-related research has been performed for researchers from Taiwan between 1991 and 2005, showing an increase of Taiwanese research articles with increasing international collaborations (7). However, a comprehensive scientometric study regarding ischemic stroke research world-wide without any restrictions such as journal type, country of publication, or publication date has not been performed up to date. This study aims at investigating the global research output on ischemic stroke research, with a focus on regional differences, differences with respect to the authors' sex, various subtopics of ischemic stroke, as well as international research collaborations.

## Methods

### Data Search

**Figure 1:**
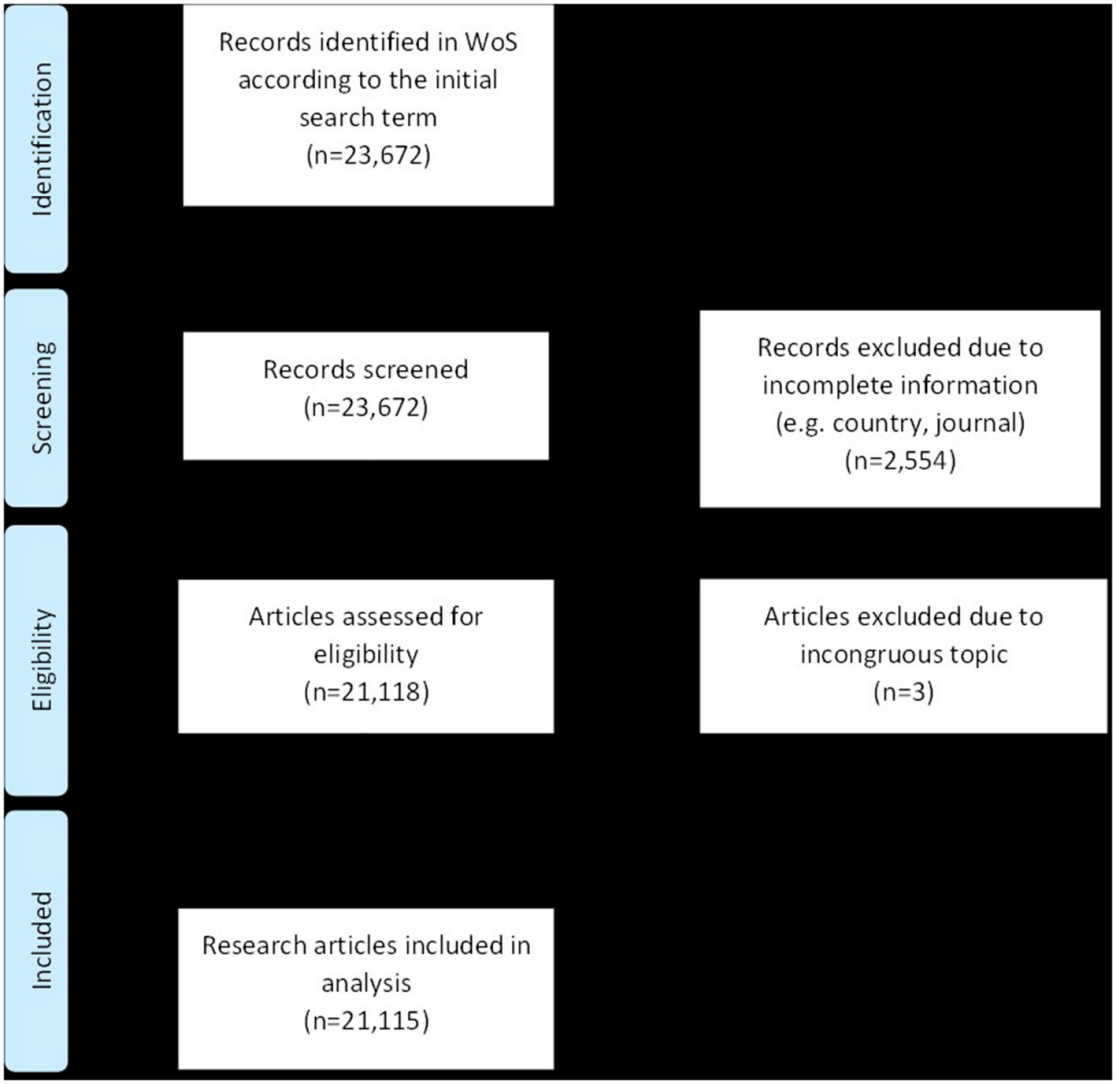
Flow chart of all included articles according to the PRISMA guidelines.

All analyzed articles were retrieved from the Web of Science Core Collection (WoS) and extracted including all respective metadata, as described in the method paper before. The search term used was a title search intended to include all original articles published by WoS on the topic of “ischemic stroke.” It read as follows: [TI = (“Isch^*^emic stroke” OR “Thrombotic stroke” OR “lacunar stroke” OR “non-lacunar stroke” OR “large artery stroke” OR “Embolic stroke” OR “cardioembolic stroke” OR “embolic stroke of undetermined source” OR ESUS OR NON-ESUS OR “embolic stroke of undetermined etiology”)]. All articles up to and including the year 2020 were included, the search was performed on 17^th^ August 2021. There were no restrictions regarding language or country of publication. In total 23,672 articles were found according to the above-mentioned search term. Of these, 2,554 articles had to be excluded as there was incomplete or missing information (e.g., regarding country of publication or type of journal). Another 3 articles had to be excluded after manual screening because the subject of the article did not match the search term. In the end, 21,115 articles remained for the final analyses. The exact search algorithm is shown in Figure 1. To minimize the likelihood of missed articles in this analysis, cross-checking with other medical databases such as PubMed was performed.

### Data Acquisition and Sex Analysis

Data were analyzed using the Science Evaluation Performance (SciPE) web tool developed by the Chair for Clinical Bioinformatics at Saarland University. SciPE was used to analyze pre-selected input metadata from WoS, as described previously (6). SciPE was specifically designed for the analysis of WoS data, as this database provides most detailed information regarding authors, country of publication, number of citations, etc. The institution-specific data analyzed here are compared to a normalized and comprehensive list of an online ranking of universities worldwide. By taking into account the geo-positioning by the Google Geocoding Service, the longitude and latitude of the institutes are used to merge the same institutes listed under different names. In the case of articles with a shared authorship, the affiliation of the first-named author is used for technical reasons. International collaborations are visualized using a chord diagram, where the width of each chord is proportional to the extent of the collaborations. In addition, all authors are classified as male, female, or unknown. The author's sex assignment was based on the author's first names, which are matched against a country-specific database. Results regarding the author's sex are only shown as percentage of female and male authors.

### H-Index

The H-index is a measure to assess the scientific performance of an author or even an institute or country. Both the number of publications and the number of citations are taken into account. The H-index describes the number of publications h that have all been cited at least h times. This index is therefore also time-dependent.

## Results

### International Publications

**Figure 2:**
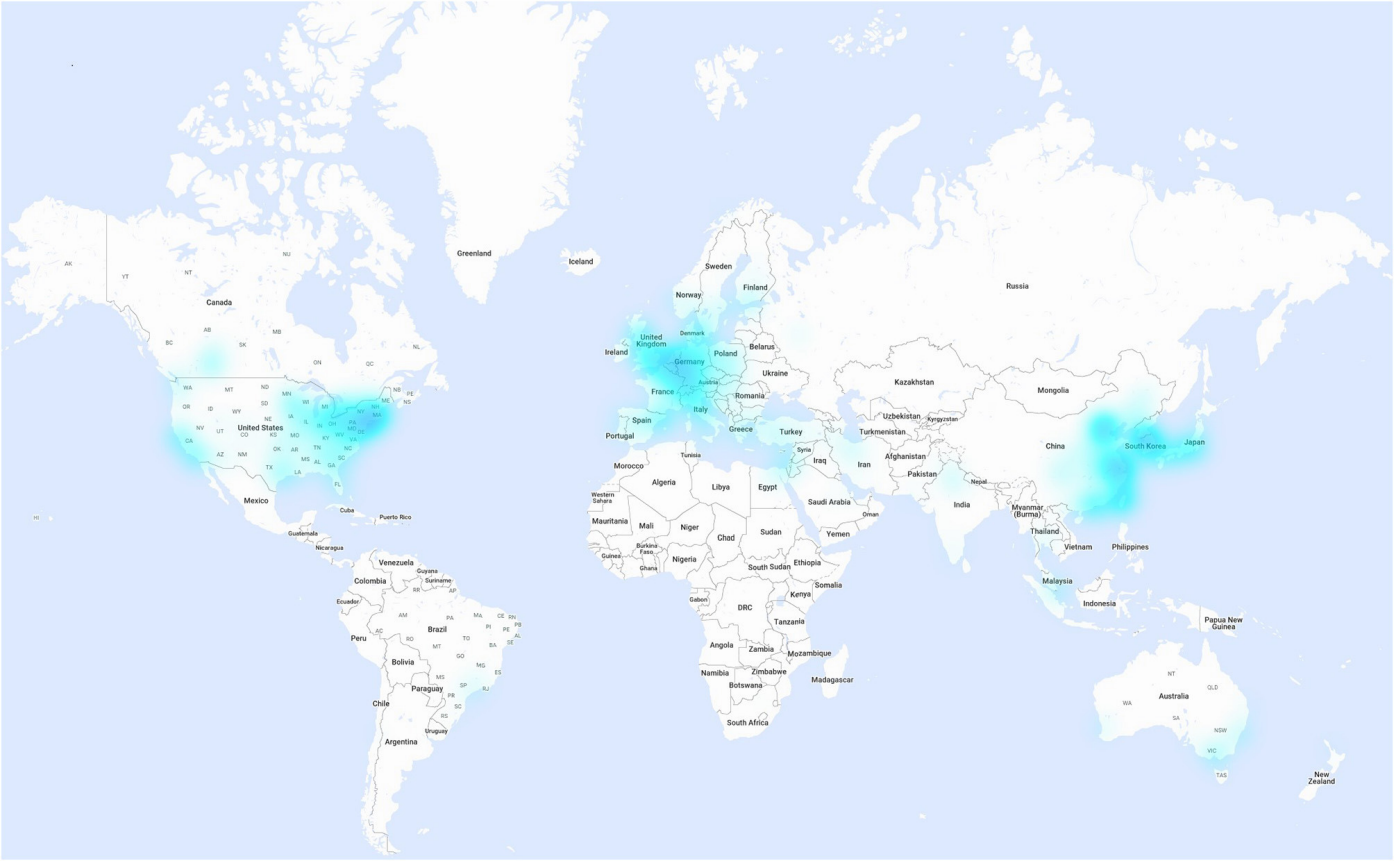
Institute heatmap of the world visualizing all research articles regarding ischemic stroke world-wide. The color intensity proportionally represents the number of publications per institute. Map data by Google [map-data, google, inegi, orion me (source https://www.google.com/maps/)].

21,115 publications regarding ischemic stroke were analyzed. Of these, 180 were published by authors from Africa, 7,644 from Asia, 7,343 from Europe, 290 from Latin America, 5,221 from North America, and 437 by authors from Oceania. A total of 132 different countries participated, with the USA contributing most publications with 4,614 (21.9%), followed by China with 3,872 (18.3%), and Germany with 1,120 (5.3%). The top 10 nations accounted with 14,925 articles for 70.7% of all publications (Table 1). The distribution of publishing institutes is visualized in the heatmap (Figure 2). Here, various regions such as the East Coast of the USA, the West Coast of China, and Central Europe are shown to be particularly productive.

**Table 1:** Characteristics of the top 20 publishing nations on ischemic stroke.

**Rank**	**Country**	**Number of publications**	**H-Index**	**Number of citations/ publication**	**% publications by female authors**
1	United States of America	4,614	202	41	27.4
2	China	3,872	81	13	34.0
3	Germany	1,120	135	49	18.8
4	Japan	998	68	17	14.7
5	South Korea	957	71	18	47.6
6	United Kingdom	791	129	50	34.9
7	Italy	760	89	26	30.1
8	Spain	646	92	31	45.8
9	Taiwan	591	60	20	44.1
10	France	576	89	28	27.7
11	Canada	529	117	49	36.3
12	Netherlands	485	86	42	53.4
13	Australia	394	91	44	29.4
14	Turkey	391	33	9	32.4
15	India	387	35	12	35.2
16	Poland	351	47	17	49.8
17	Switzerland	268	73	32	26.1
18	Sweden	248	73	37	45.6
19	Denmark	220	56	29	47.9
20	Russia	217	24	6	54.3
	Mean	921	83	29	36.8%

### Publication Quality According to H-Index and Citations

**Figure 3:**
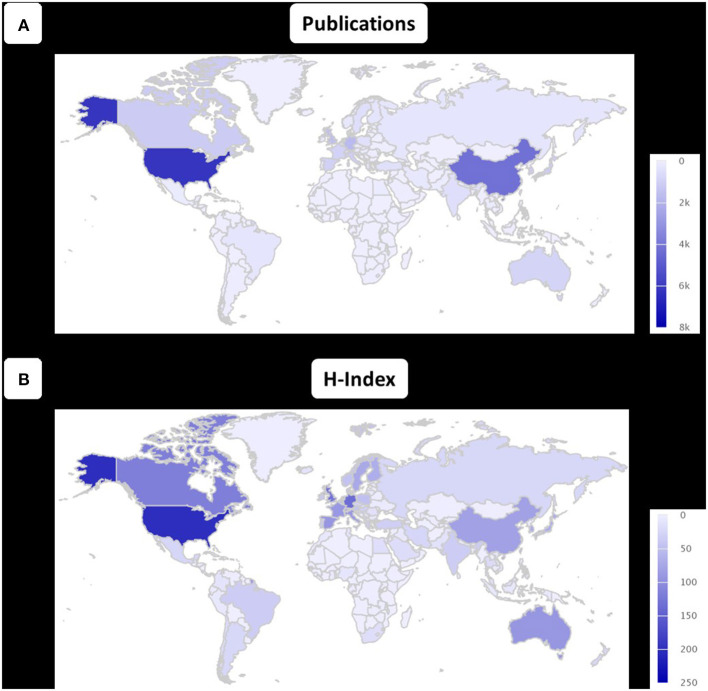
Global distribution of number of publications and H-index. World map showing the number of publications **(A)** and the H-index **(B)** of research in ischemic stroke. The legend on the right side codes the color intensity of the map.

When analyzing the scientific quality of different countries by H-index, the USA ranked first with an H-index of 202, followed by Germany (H-index 135) and the United Kingdom (UK; H-index 129) (Figure 3). Comparing the average number of citations per article, the UK ranked first with an average of 50.27 citations per publication, followed by Canada (49.35) and Germany (49.28).

### Publication Distribution Over Time

There has been a substantial increase in the number of publications over the years. About 4% of all publications were published before 2000 (829 of the total 21,115 publications), while 18.7% (3,942 of 21,115 publications) were published between 2000 and 2010. Thus, the majority, 77.4% (16,345 of 21,115), was published after 2010.

### Topics

All publications were automatically categorized into topics by the WoS database. The most frequently used topic was “Clinical Neurology” with 9,028 publications, followed by “Neurosciences” with 1,920 publications. “General and Internal Medicine” with 1,857, “Cardiac & Cardiovascular Systems” with 1,248, and “Peripheral Vascular Disease” with 612 publications follow in 3^rd^ to 5^th^ place.

### Sex Distribution

Among all first authors that were attributed to their sex, 32.3% of all first authors were female and 67.7% were male (4,335 vs. 9,097). The proportion of female last authors was comparatively lower at 22.4% (3,083 publications) compared with 77.6% male authors (10,658 publications). The author's sex remained unknown in 36.4% of all cases. Among the top 20 most published countries on the topic of ischemic stroke, the countries with the largest proportion of female authors were Russia (54.3%), the Netherlands (53.4%), and Poland (49.8%). In contrast, the lowest proportion of women was found in Japan (14.7%), Germany (18.8%), and Switzerland (26.1%) (Table 1), respectively.

### International Collaborations

**Figure 4:**
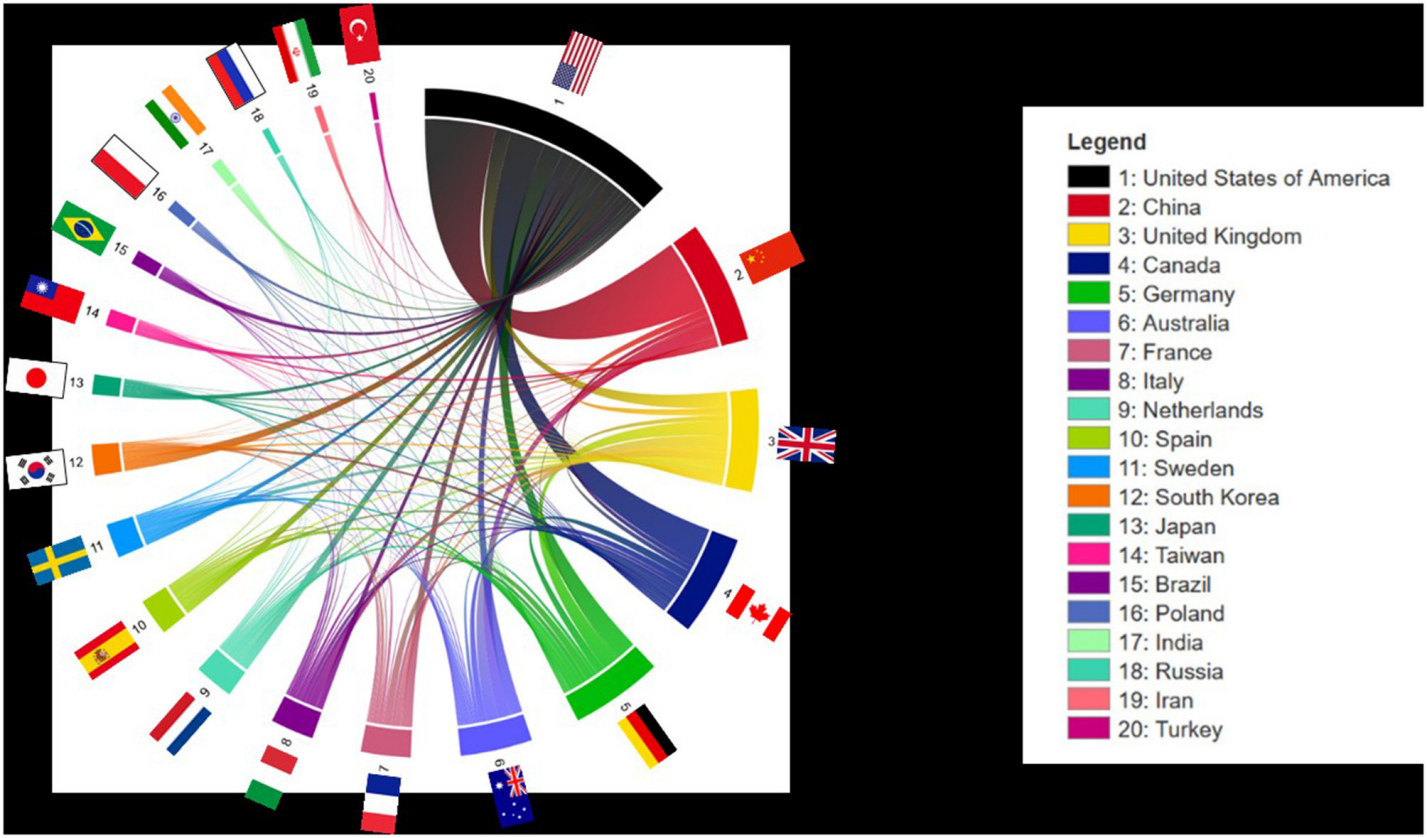
Chord diagram of international collaborations. Visualization of all countries with international collaborations sorted by frequency of global collaboration. The connection chords of the chord diagram reflect the frequency of joint publications.

There was a broad network of international collaborations. The USA represented the country with most collaborations on the topic of ischemic stroke. The most frequent collaborator was China, followed by the UK, Canada and Germany. The chord diagram in Figure 4 shows the collaborations of the 20 most published countries on the research area of ischemic stroke.

## Discussion

This is the first scientometric analysis on global ischemic stroke research output. Of all 21,115 scientific articles analyzed, the United States contributed the most with 4,614 articles (21.9%). At the same time, the USA lead the publication quality measured by the H-index with 202. This is in line with previous scientometric analyses on cardiovascular diseases, in which the USA were the leading nation in terms of research output (8). The second place in the absolute number of publications is occupied by China with 3,872 articles, corresponding to 18.3% of all analyzed papers. Interestingly, as previously mentioned, differences in publication quality and quantity of research work from Asian authors (9) were again visible in the present study. While ranking second in terms of number of publications, China ranked 10^th^ in research quality as measured by its H-index. Consistent with the growing prevalence and incidence of stroke world-wide (10), a relevant increase in research articles could be seen as well. However, the increase in scientific articles on the topic of ischemic stroke may not only be due to the increasing burden of strokes, as there has also been a general increase in publications in the field of cardiovascular disease, so this increase may not only be stroke-specific (8, 9).

Research on ischemic stroke has been published primarily in neurological journals because of the neurological nature of the disease. Accordingly, the WoS also classified the research into these topics, such as “Clinical Neurology” (42.8% of all publications) and “Neurosciences” (9.1%). Nevertheless, some articles on the field of ischemic stroke have also been placed in journals that are not exclusively neurological, as shown by the subsequent topics being “General & Internal Medicine,” “Cardiac & Cardiovascular Systems,” and “Peripheral Vascular Diseases” (8.8, 5.9, and 2.9%, respectively). These topics indicate the interdisciplinary nature of the disease “ischemic stroke,” with a strong interconnectedness also in the field of internal medicine, especially cardiovascular medicine. The reason for this is to be found in particular in the causes of ischemic stroke, since ESUS, for example, are often triggered by-mostly still undetected-cardiovascular diseases, such as subclinical atrial fibrillation, a persistent foramen ovale (PFO), but also valvular heart disease and other cardiovascular diseases (11).

In the present analysis, the proportion of female first authors was with 32.3% substantially lower than that of male first authors with 67.7% (4,332 vs. 9,097 articles, respectively). This difference was even greater when comparing the last authors (22.4% female vs. 77.6% male). This sex difference in authors of ischemic stroke related articles has already been described among authors of scientific papers in other fields, such as cardiovascular medicine (8). Here, the difference was consistently seen in the last decade, but the proportion of female authors-both first and last authors-was increasing (8).

The research landscape around ischemic stroke features extensive scientific networks worldwide. The collaborations shown in Figure 4 using a chord diagram indicates the extent of these collaborations. Interestingly, the USA is the country with most collaborations, as well as the leading nation in terms of the absolute number of publications. China-the country with the second most published articles-recorded a fewer number of cooperation partners. However, Chinese scientists are the most frequent collaborating partners for researchers from the USA, followed by the UK, Canada, and Germany. It has already been shown that cross-border collaborations had a greater impact on citations than domestic publications (12). This is in line with the higher H-index measured in the field of ischemic stroke for the USA as a highly collaborative country, compared to China.

### Limitations

There are some limitations that need to be addressed in this citation-based study. Only articles published at WoS were considered, due to the technical requirements of the SciPE analysis tool. Despite careful cross-checking with other medical databases such as Pubmed, it cannot be ruled out that some articles were not included. Furthermore, the results depend on the search term. However, the search results were designed to be as accurate as possible through thorough testing in advance and assessment of eligibility of the search results afterwards. In the analysis of country affiliation or sex assignment, only the first-mentioned author was considered. Thus, in the rare case of a shared first authorship, the second-named first author was not considered. As already mentioned, sex assignment was performed by analyzing the first name of an author. Even though country-specific differences were taken into account (“Andrea” female for German authors, but male for Italian ones), there was still a part that could not be assigned. This was also the case when only the initial was given instead of the first name. However, an automated analysis is a requirement for the implementation of such a large-scale analysis.

## Conclusion

Research in ischemic stroke has substantially increased over time. Scientists from the USA have the highest number of publications, followed by China and Germany. Measured by the H-index, the USA was able to demonstrate the highest publication quality, followed by Germany and the UK. The topic of ischemic stroke has many interfaces with cardiovascular medicine, which is particularly evident in the search for underlying causes. The scientific landscape is predominantly male, especially among the last authors. Worldwide international collaborations play a major role in ischemic stroke research.

## Data Availability Statement

The raw data supporting the conclusions of this article will be made available by the authors, without undue reservation.

## Author Contributions

DM made substantial contributions to the conception of the work, analyzed and interpreted the data for the work, and drafted the manuscript. AR-S, PL, MB, and KF gave substantial contribution to the concept of the work and revised the manuscript critically for important intellectual content. TF and MR made substantial contributions to the acquisition of the data and revised the manuscript critically for important intellectual content. AK and FM made substantial contributions to the interpretation of the data. Both revised the manuscript critically for important intellectual content. CU made substantial contributions to the conception and design of the work as well as the interpretation of the data, drafted, and revised the manuscript critically for important intellectual content. All authors reviewed and edited the manuscript and approved the final version of the manuscript.

## Funding

MB and FM are supported by Deutsche Forschungsgemeinschaft (SFB TRR219). FM is supported by Deutsche Gesellschaft für Kardiologie (DGK).

## Conflict of Interest

DM has received honoraria from Bayer, Boston Scientific, and Daiichi Sankyo. MB receives honoraria for lectures and scientific advice from Abbott, Astra-Zeneca, Boehringer-Ingelheim, Medtronic, Novartis, Servier, and Vifor. FM has received scientific support and speaker honoraria from Bayer, Boehringer Ingelheim, Medtronic, and ReCor Medical. CU received scientific support and speaker honorarium from Bayer, Boehringer-Ingelheim, Medtronic Inc., Recor Medical, and Pfizer. The remaining authors declare that the research was conducted in the absence of any commercial or financial relationships that could be construed as a potential conflict of interest.

## Publisher's Note

All claims expressed in this article are solely those of the authors and do not necessarily represent those of their affiliated organizations, or those of the publisher, the editors and the reviewers. Any product that may be evaluated in this article, or claim that may be made by its manufacturer, is not guaranteed or endorsed by the publisher.

## Abbreviations

ESUS: Embolic stroke of undetermined source
H-Index: Hirsch-Index
PFO: Patent foramen ovale
SciPE: Science Performance Evaluation
USA: United States of America
vs.: versus
WoS: Web of Science Core Collection.
